# Pretreatment lymphopenia is an easily detectable predictive and prognostic marker in patients with metastatic esophagus squamous cell carcinoma receiving first‐line chemotherapy

**DOI:** 10.1002/cam4.638

**Published:** 2016-01-26

**Authors:** Furong Kou, Zhihao Lu, Jian Li, Xiaotian Zhang, Ming Lu, Jun Zhou, Xicheng Wang, Jifang Gong, Jing Gao, Jie Li, Yan Li, Lin Shen

**Affiliations:** ^1^ Key laboratory of Carcinogenesis and Translational Research (Ministry of Education)Department of GI OncologyPeking University School of OncologyBeijing Cancer Hospital & InstituteBeijing100142China

**Keywords:** Chemotherapy, efficacy, esophageal squamous cell carcinoma, lymphopenia, prognosis, toxicity

## Abstract

To explore the influence of pretreatment lymphopenia on the toxicity and efficacy of first‐line chemotherapy in patients with metastatic esophagus squamous cell carcinoma (ESCC). In total, 215 patients were included in this retrospective study. Correlations between pretreatment lymphopenia (lymphocyte count <1 × 10^9^/L) and the occurrence of toxicity and the efficacy of first‐line palliative chemotherapy were investigated. Pretreatment lymphopenia was found in 19.1% of the patients. The overall response rate (ORR) was 35.5% (65 of 183 patients). Patients with pretreatment lymphopenia had a lower ORR to chemotherapy compared with those without lymphopenia (22.2% vs. 38.8%, respectively; *P *=* *0.045). Furthermore, the patients with pretreatment lymphopenia have higher grade 3–4 hematological toxicity than that of patients without pretreatment lymphopenia (19 of 41 patients, 46.3% vs. 54 of 174 patients, 31.0%; *P *=* *0.048). Pretreatment lymphopenia was not correlated with grade 3–4 nonhematological toxicity. Multivariate analysis showed that pretreatment lymphopenia is an independent prognostic factor. Patients with pretreatment lymphopenia had a significantly shorter overall survival time than those without lymphopenia (8.2 months vs. 12.7 months; *P *=* *0.020). This study shows that pretreatment lymphopenia is a good prognostic factor as well as a predictive factor for tumor response and chemotherapy‐related hematological toxicity in metastatic ESCC.

## Introduction

Esophageal carcinoma is one of the most common cancers in the world, with an incidence that ranks eighth among all of the malignant cancers 1. In contrast with the predominant adenocarcinoma in western countries, ~95% of esophageal carcinoma in China can be classified as esophagus squamous cell carcinoma (ESCC). Most patients were diagnosed at the advanced stage, yet, treatment is mainly palliative and based on systemic chemotherapy.

The combination of cisplatin and 5‐fluorouracil was considered the standard regimen, with an efficacy of 33–35% 2, 3. Unfortunately, new cytotoxic drugs (paclitaxel, docetaxel, irinotecan, etc.) 4, 5, 6 and targeted drugs (cetuximab 7, gefitinib 8, etc.) did not make a breakthrough with regard to efficacy and improving survival of patients with ESCC. Moreover, chemotherapy‐related toxicities, especially hematological toxicities such as leukopenia, neutropenia, and febrile neutropenia were common. The rate of grade 3–4 hematological toxicity was 14–34.1% when treated with cisplatin, 5‐fluorouracil, docetaxel, or paclitaxel 2, 3, 5, 6. In one study, febrile neutropenia was also found in 6.1% patients 5.

It is necessary to screen patients who are most likely to benefit from chemotherapy with high efficacy and low toxicity, which may lead to a good prognosis. Previously, too much attention was focused on the tumor itself. Cancer is a systemic disease, and the immune system of the host is thought to play a central role in cancer suppression 9. Lymphocytes are crucial components of the immune system that may affect tumor growth and the survival of patients. Recently, pretreatment lymphopenia has been shown to be a poor prognostic factor for various cancers 10, 11, 12, 13, 14, 15. Furthermore, lymphopenia has been shown to be a powerful predictor of chemotherapy‐induced toxicity, as well as of the efficacy of chemotherapy in colorectal cancer, breast cancer, lung cancer, etc. 10, 16, 17, 18. The overall response rate (ORR) in patients with pretreatment lymphopenia was significantly lower than in patients with normal lymphocyte counts 10, 16, 17, 18. However, no studies focused on metastatic ESCC patients receiving first‐line chemotherapy.

Therefore, the purpose of this retrospective study was to examine the impact of pretreatment lymphocyte counts on survival, tumor response, and treatment‐related toxicity in metastatic ESCC patients receiving first‐line chemotherapy.

## Patients and Methods

### Patients

This study is a retrospective analysis of a cohort of patients with esophageal cancer at the Peking University Cancer Hospital Gastrointestinal Medical Oncology Department between January 2005 and January 2013. Detailed clinical data for patients were recorded in a regularly updated electronic database. Eligibility criteria included the following: (1) all pathologically confirmed ESCC with metastatic diseases (the 7th edition of the American Joint Committee on Cancer Staging); (2) patients receiving first‐line chemotherapy; (3) life expectancy ≥3 months; and (4) pretreatment and follow‐up laboratory values measured at our institution are available in the electronic medical record. Patients were excluded if they fulfilled the following criteria: (1) they experienced recurrence within 6 months after adjuvant chemotherapy or chemoradiation therapy; or (2) they had pathologically confirmed esophageal adenocarcinoma, small cell carcinoma, lymphoma, or adenosquamous cell carcinoma. The study was approved by the Research Ethics Committee of Peking University Cancer Hospital.

### Data collection

Demographic, treatment, and laboratory‐based characteristics were obtained from the electronic medical records of each patient. Patient‐specific variables included age, sex, Karnofsky Performance Status (KPS), height, weight, and weight loss before chemotherapy. Tumor‐specific variables included primary tumor location, histologic grade, and sites of metastasis. Treatment parameters consisted of regimens of first‐line chemotherapy, second‐line therapy, radiation, and whether or not patients received surgery. Pretreatment laboratory values, including complete blood counts (white blood cell, neutrophil, lymphocyte, platelet values), albumin, and tumor makers (CEA, CYFRA2‐11 and SCC), were recorded before first‐line chemotherapy was administered. The dichotomization of these variables was based on the upper (white blood cells, neutrophils, platelet, and tumor markers) and lower (albumin and lymphocytes) ranges of the normal measurements for these markers. Neutrophil–lymphocyte ratio (NLR) and platelet–lymphocyte ratio (PLR) were also calculated and defined as variables for analysis. We used the medians of NLR and PLR as cutoff points for dichotomization.

### Assessment of toxicity, response and survival

Hematological toxicity and nonhematological toxicity were recorded according to the National Cancer Institute Common Toxicity Criteria Version 3.0 (NCI‐CTC.V3.0) based on direct questioning, physical examination, and laboratory tests. All patients who had received at least one course of chemotherapy were evaluated for toxicity. Tumor assessment was performed every 6 weeks or earlier in cases of clinical suspicion of progression using computed tomography scanning. The objective response to treatment was classified using the Response Evaluation Criteria in Solid Tumors (RECIST 1.0). For this analysis, patients with complete or partial response were classified as responders, and those with stable or progressive disease were classified as nonresponders. ORR was the percentage of responders of the total patients. Overall survival (OS) was calculated from the first day of chemotherapy to death from any cause or last follow‐up, at which time data were censored. Survival data were available for all patients.

### Statistical analysis

**Figure 1:**
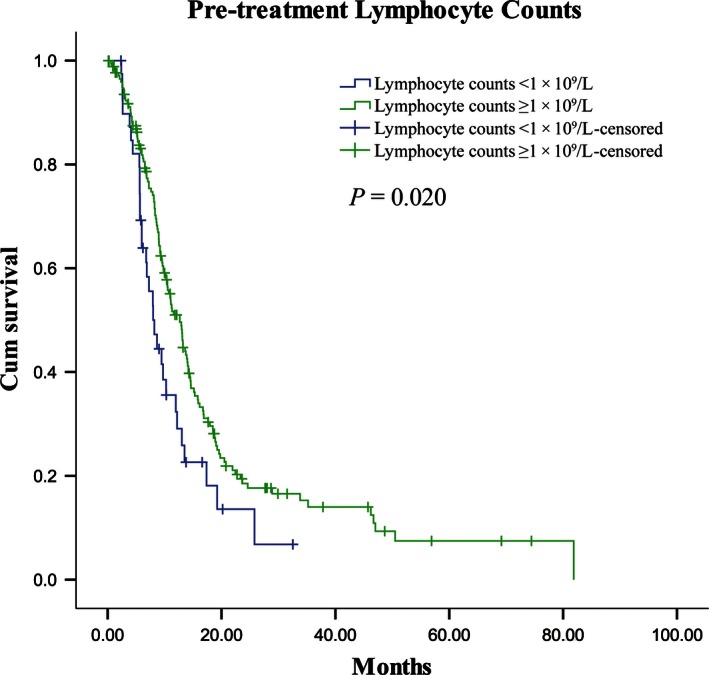
Kaplan–Meier curves of overall survival (OS) according to pretreatment lymphocyte counts. Patients with pretreatment lymphocyte counts <1 × 10^9^/L had shorter OS (median 12.7 months, *n* = 41) than patients with lymphocyte counts ≥1 × 10^9^/L (median 8.2 months, *n* = 174; *P *=* *0.020).

Different potential predictive variables for OS were considered to be dichotomous. Accordingly, pretreatment lymphocyte count was considered to be <1 × 10^9^/L (defined as lymphopenia) or ≥1 × 10^9^/L (Fig. 1). The chi‐squared test was used to compare proportions between groups. Univariate survival analysis was performed using the Kaplan–Meier method with the log‐rank test. Multivariate survival analysis was performed using a Cox regression model including those factors that were significant (*P *<* *0.1) on univariate analysis. SPSS (version 17.0, Inc., Chicago, IL) statistical software was used for the statistical analysis. All *P*‐values were two sided, and *P *<* *0.05 was considered statistically significant.

## Results

### Patient characteristics

From January 2005 to January 2013, 215 patients were eligible for this retrospective analysis. The last follow‐up time was April 1, 2015. Demographic, treatment and pretreatment laboratory characteristics of these patients are summarized in Table 1.

**Table 1:** Patient characteristics (*N* = 215)

Clincopathological characteristics	*N* (%)
Median age (years) [range]	58 [42–82]
Gender: male vs. female	184 (85.6) vs. 31 (14.4)
KPS: >80 vs. ≤80	151 (70.2) vs. 64 (29.8)
Weight loss: ≤5% vs. >5%	132 (61.4) vs. 83 (38.6)
Grade: well or moderately vs. poorly or undifferentiated	112 (52.1) vs. 103 (47.9)
Primary tumor location: cervical or upper vs. middle or lower	36 (16.7) vs. 179 (83.3)
Liver metastasis	61 (28.4)
Lung metastasis	74 (34.4)
Bone metastasis	20 (9.3)
Distant lymph node metastasis	171 (79.5)
Number of metastatic sites: <3 vs. ≥3	175 (81.4) vs. 40 (18.6)
First‐line chemotherapy: PTX‐based regimen vs. non‐PTX‐based regimens^a^	160 (74.4) vs. 55 (25.6)
Therapy after first‐line chemotherapy	137 (63.7)
Second‐line chemotherapy	47 (21.9)
PTX‐based chemotherapy	18 (8.3)
Irinotecan‐based chemotherapy	24 (11.2)
Other chemotherapy regimens^b^	5 (2.3)
Palliative radiotherapy	120 (55.8)
Other treatments^b^	5 (2.3)
Surgery history	76 (35.3)
Radiation: radical vs. palliative^b^	10 (4.7) vs. 125 (58.1)
White blood cell count:>10 × 10^9^/L [range]	19 (8.8) [2.91–18 × 10^9^/L]
Neutrophil count: >8 × 10^9^/L [range]	14 (6.5) [0.84–14.73 × 10^9^/L]
Lymphocyte count: <1 × 10^9^/L [range]	41 (19.1) [0.3–5.14 × 10^9^/L]
Platelet count: >300 × 10^9^/L [range]	35 (16.3) [81–615 × 10^9^/L]
NLR: median [range]	3.0 [0.47–12.79]
PLR: median [range]	153.0 [19.64–723.33]
Albumin: ≤40 g/L [range]	42 (19.5) [18.1–51.5 g/L]
CEA: >5 ng/mL [range]	45 (20.9) [0.2–994.2 ng/mL]
CYFRA2‐11: >3.3 ng/mL [range]	102 (47.4) [0.8–237.3 ng/mL]
SCC: >1.5 ng/mL [range]	91 (42.3) [0–96 ng/mL]

KPS, Karnofsky Performance Status; PTX, Paclitaxel; NLR, neutrophil‐lymphocyte ratio; PLR, platelet‐lymphocyte ratio.

a Non‐PTX‐based regimens: fluorouracil‐based, irinotecan‐based, cisplatin‐based, gemcitabine‐based, capecitabine‐based, S‐1‐based and etoposide‐based.

b Other chemotherapy regimens: fluorouracil‐based, cisplatin‐based, gemcitabine‐based, etoposide‐based, oxaliplatine‐based and S‐1‐based. Other treatments: third‐line chemotherapy, transarterial chemoembolization, radiofrequency ablation. Palliative radiation fields: brain, bone, lung, liver, lymph node, and anastomotic stoma.

The median age was 58 years with a range of 42–82. The majority of patients were male (85.6%). All patients presented with metastases, while 61 (28.4%) patients were afflicted with liver, 74 (34.4%) with lung, and 20 (9.3%) with bone metastasis. Other metastatic sites, such as brain, adrenal glands, kidney, and pleura, were rare. All patients received first‐line chemotherapy, with 74.4% of the patients receiving a paclitaxel‐based regimen. Other drugs in non‐paclitaxel‐based regimens included fluorouracil, irinotecan, and cisplatin etc. A small proportion of patients (24.2%) used nimotuzumab‐targeted therapy during chemotherapy. Ten (4.7%) patients received radical esophagus radiation, while palliative radiation was applied in 125 (58.1%) patients. The targets of palliative radiotherapy included the primary tumor or the sites of metastasis (brain, bone, lung, liver, lymph node, etc.). After first‐line chemotherapy, 137 (63.7%) patients received subsequent therapy, including second‐line chemotherapy (47 patients), palliative radiotherapy (120 patients), and other treatments (5 patients). Most patients had normal pretreatment complete blood count values, while 19.1% of the patients had pretreatment lymphopenia. The median values of NLR and PLR were 3.0 and 153.0.

### Univariate and multivariate analyses of prognostic factors for OS

The median OS for the entire cohort (*N* = 215) was 11.1 months (95% CI, 8.982–13.218). Among the 26 variables in the univariate analysis, 15 variables were identified to be statistically significantly prognostic factors (gender, weight loss, liver metastasis, bone metastasis, number of metastatic sites, first‐line chemotherapy, second‐line chemotherapy, palliative radiation history, white blood cell count, neutrophil count, lymphocyte count, NLR, PLR, CYFRA2‐11, SCC; *P *<* *0.05).

The 17 factors identified in the above‐described univariate analysis (*P *<* *0.1) were used to construct the multivariate cox proportional hazards model for survival. The following eight factors remaining in the model were considered independent prognostic factors: gender (*P *=* *0.000), weight loss (*P *=* *0.000), liver metastasis (*P *=* *0.026), first‐line chemotherapy (*P *=* *0.000), second‐line chemotherapy (*P *=* *0.000), surgery history (*P *=* *0.000), palliative radiation history (*P *=* *0.000), and pretreatment lymphopenia (*P *=* *0.021). The results of univariate and multivariate analyses are shown in Table 2.

**Table 2:** Univariate and multivariate analysis of the characteristics associated with the overall survival

Characteristics	mOS (month)	Univariate analysis		Multivariate analysis	
		Chi‐square	*P* value	HR (95% CI)	*P* value
Clinicopathological characteristics					
Age (≤65/>65)	11.1/12.0	0.001	0.970		
Gender (Male/Female)	10.3/28.9	10.651	**0.001**	0.298 (0.162–0.549)	**0.000**
KPS (>80/≤80)	11.1/11.2	0.229	0.632		
Weight loss (≤5%/>5%)	13.5/9.0	16.641	**0.000**	1.991 (1.363–2.909)	**0.000**
Grade (well or moderately/poorly or undifferentiated)	12.0/11.1	0.206	0.650		
Primary tumor location (Cervical or upper/Middle or lower)	13.5/10.7	0.937	0.333		
Liver metastasis (Yes/No)	8.7/12.9	14.178	**0.000**	1.559 (1.053–2.307)	**0.026**
Lung metastasis (Yes/No)	10.6/13.0	0.021	0.886		
Bone metastasis (Yes/No)	9.0/11.3	4.123	**0.042**		
Distant lymph node metastasis (Yes/No)	10.5/13.1	0.086	0.770		
Number of metastatic sites (<3/≥3)	12.0/8.7	6.745	**0.009**		
Treatment characteristics					
First‐line chemotherapy (PTX‐based regimen/non‐PTX‐based regimens)	13.0/8.1	20.616	**0.000**	0.366 (0.244–0.549)	**0.000**
Second‐line chemotherapy (Yes/No)	15.8/9.5	5.416	**0.020**	0.474 (0.315–0.713)	**0.000**
Surgery history (Yes/No)	13.5/10.6	3.185	**0.074**	0.486 (0.330–0.717)	**0.000**
Radical radiation history (Yes/No)	12.0/11.1	0.322	0.571		
Palliative radiation history (Yes/No)	13.9/8.6	13.264	**0.000**	0.475 (0.332–0.680)	**0.000**
Pretreatment laboratory characteristics					
White blood cell count (≤10/>10)×10^9^/L	11.3/8.7	6.374	**0.012**	1.711 (0.934–3.134)	0.082
Neutrophil count (≤8/>8)×10^9^/L	11.3/5.2	8.281	**0.004**		
Lymphocyte count (<1/≥1)×10^9^/L	12.7/8.2	5.435	**0.020**	0.586 (0.373–0.922)	**0.021**
Platelet count (≤300/>300)×10^9^/L	11.2/8.7	3.186	**0.074**		
NLR (≤3.0/>3.0)	13.5/8.7	13.337	**0.000**		
PLR (≤153.0/>153.0)	13.6/9.5	7.879	**0.005**		
Albumin (≤40/>40) g/L	11.3/11.1	0.641	0.423		
CEA (≤5/>5) ng/mL	11.3/10.5	0.400	0.527		
CYFRA2‐11 (≤3.3/>3.3) ng/mL	13.7/9.8	8.946	**0.003**		
SCC (≤1.5/>1.5) ng/mL	13.7/9.8	13.705	**0.000**	1.422 (0.972–2.081)	0.069

mOS, median overall survival; HR, hazard ratio; KPS, Karnofsky Performance Status; PTX, Paclitaxel; NLR, neutrophil‐lymphocyte ratio; PLR, platelet‐lymphocyte ratio. Bold values indicate a statistically difference in univariate analysis (*P*< 0.1) and statistically significant difference in multivariate analysis (P<0.05).

### Relationship between pretreatment lymphopenia and patient characteristics

Pretreatment lymphopenia was significantly correlated with liver metastasis (*P *=* *0.005), bone metastasis (*P *=* *0.019), and number of metastatic sites (*P *=* *0.000). As for the other clinicopathological data (age, sex, KPS, weight loss, primary tumor location, lung metastasis, and distant lymph node metastasis), no significant differences were detected between the groups. Moreover, pretreatment lymphopenia was also significantly correlated with some laboratory data, including white blood cell count, neutrophil count, NLR, and PLR. There were no significant differences in treatment‐related data between the groups except for surgery history (*P *=* *0.001). The relationships between the pretreatment lymphocyte counts and patients characteristics are shown in Table 3.

**Table 3:** Comparison of the characteristics and treatment efficacy of patients with or without pretreatment lymphopenia

	Patients with lymphocyte count		Chi‐square	*P* value
	<1 × 10^9^/L (*n* = 41)	≥1 × 10^9^/L (*n* = 174)		
Clinicopathological characteristics				
Age (≤65/>65)	28/13	133/41	1.170	0.188
Gender (Male/Female)	38/3	146/28	2.070	0.133
KPS (>80/≤80)	29/12	122/52	0.006	0.551
Weight loss (≤5%/>5%)	25/16	107/67	0.004	0.543
Grade (well or moderately/poorly or undifferentiated)	23/16	89/77	0.366	0.366
Primary tumor location (cervical or upper/middle or lower)	7/34	29/145	0.004	0.555
Liver metastasis (Yes/No)	19/22	42/132	8.049	**0.005**
Lung metastasis (Yes/No)	16/25	58/116	0.476	0.303
Bone metastasis (Yes/No)	8/33	12/162	6.259	**0.019**
Distant lymph node metastasis (Yes/No)	31/10	140/34	0.480	0.31
Number of metastatic sites (<3/≥3)	24/17	151/23	17.481	**0.000**
Treatment characteristics				
First‐line chemotherapy (PTX‐based regime/non‐PTX‐based regimes)	27/14	133/41	1.952	0.117
Second‐line chemotherapy (Yes/No)	7/35	41/133	0.931	0.227
Surgery history (Yes/No)	24/17	52/122	11.919	**0.001**
Radical radiation history (Yes/No)	4/37	6/168	2.977	0.100
Palliative radiation history (Yes/No)	23/18	72/102	2.915	0.063
Pretreatment laboratory characteristics				
White blood cell count (≤10/>10) ×10^9^/L	41/0	155/19	4.793	**0.016**
Neutrophil count (≤8/>8)×10^9^/L	41/0	160/14	3.444	**0.050**
Platelet count (≤300/>300)×10^9^/L	35/6	146/28	0.053	0.517
NLR (≤3.0/>3.0)	3/38	104/70	36.518	**0.000**
PLR (≤153.0/>153.0)	2/39	105/69	40.835	**0.000**
Albumin (≤40/>40) g/L	10/31	32/142	0.760	0.252
CEA (≤5/>5) ng/mL	29/12	139/33	2.020	0.115
CYFRA2‐11 (≤3.3/>3.3) ng/mL	14/22	72/80	0.843	0.233
SCC (≤1.5/>1.5) ng/mL	24/15	89/76	0.737	0.249
Treatment efficacy				
CR + PR/SD + PD	8/28	57/90	3.460	**0.045**
ORR[(CR + PR)%]	22.2%	38.8%		

KPS, Karnofsky Performance Status; PTX, Paclitaxel; NLR, neutrophil‐lymphocyte ratio; PLR, platelet‐lymphocyte ratio; CR, complete response; PR, partial response; SD, stable disease; PD, progressive disease; ORR, overall response rate. Bold values indicate statistically significant difference (*P*≤0.05) in chi‐square test.

### Tumor response and pretreatment lymphocyte counts

Of the total 215 patients, 183 patients were evaluated for response to first‐line chemotherapy. The ORR was 35.5% (65 of 183 patients). Pretreatment lymphopenia was significantly associated with lower ORR to chemotherapy. Eight of the 36 patients with pretreatment lymphopenia responded to chemotherapy versus 57 of the 147 patients without pretreatment lymphopenia (22.2% vs. 38.8%, respectively; *P *=* *0.045). The details are summarized in Table 3.

### Treatment‐related toxicities and pretreatment lymphocyte counts

The most common hematological toxicities were leukopenia (67.4%) and neutropenia (61.9%). Similarly, leukopenia and neutropenia were also the most common grade 1–2 and grade 3–4 toxicities (45.6% and 30.2%, respectively). Moreover, the total hematological toxicity for all patients was 22.3% (48 patients) for grade 3 and 11.6% (25 patients) for grade 4. The most prevalent nonhematological toxicity was nausea (67.0%), with 62.3% of cases being grade 1–2 and 4.7% being grade 3–4.

The relationship between pretreatment lymphopenia and grade 3–4 toxicity was assessed in 215 patients (Table 4). Grade 3–4 hematological toxicity was observed in 19 of 41 patients with pretreatment lymphopenia (46.3%), and in 54 of 174 patients (31.0%) with lymphocyte count ≥1 × 10^9^/L (*P *=* *0.048). Pretreatment lymphopenia was not correlated with grade 3–4 nonhematological toxicity (including fatigue, nausea, vomiting, diarrhea, hair loss, muscle/joint pain, and liver damage).

**Table 4:** Comparison of the grade 3–4 hematological and nonhematological toxicities of patients with or without pretreatment lymphopenia (*N* = 215)

Grade 3–4 toxicity	Patients with lymphocyte count, *n* (%)		Chi‐square	*P* value
	<1 × 10^9^/L (*n* = 41)	≥1 × 10^9^/L (*n* = 174)		
Hematological toxicity	19 (46.3)	54 (31.0)	3.467	**0.048**
Fatigue	1 (2.4)	5 (2.9)	0.023	0.687
Nausea	1 (2.4)	9 (5.2)	0.559	0.399
Vomiting	1 (2.4)	8 (4.6)	0.386	0.461
Diarrhea	0	2 (1.1)	0.476	0.654
Hair loss	0	10 (5.7)	2.471	0.115
Muscle/joint pain	0	1 (0.6)	0.237	0.809
Liver damage	1 (2.4)	6 (3.4)	0.107	0.602

Bold values indicate statistically significant difference (*P*≤0.05) in chi‐square test.

## Discussion

In this study, we established a prognostic model for metastatic ESCC patients receiving first‐line chemotherapy consisting of eight factors: pretreatment lymphopenia, gender, weight loss, liver metastasis, first‐line chemotherapy regimens, second‐line chemotherapy, primary tumor surgery, and palliative radiation. Furthermore, pretreatment lymphopenia was shown to be not only as a prognostic factor for short‐term survival but also as a predictive factor for tumor response and treatment‐related hematological toxicity in these patients.

To the best of our knowledge, the host immune system plays a central role in cancer suppression 9. Various factors are related to the body's immune status, such as lymphocytes in the peripheral blood, tumor‐infiltrating lymphocytes (TILs) 19, 20, 21, and treatment‐related lymphopenia 12, 22, 23. Lymphocytes in the peripheral blood are considered to be crucial components of the immune system and to reflect immune responsiveness. Lymphopenia, which was associated with an immunosuppressed state, was common in patients with solid tumors 10, 11, 12, 24. Previous studies have demonstrated that lymphopenia is related to the short‐term survival of various cancers, such as lung cancer, glioma, colorectal cancer, pancreatic cancer, and nasopharyngeal cancer 10, 11, 12, 13, 14, 18. Feng et al. 15 also found that preoperative lymphopenia is an independent poor prognostic factor in ESCC patients who had undergone esophagectomy without preoperative neoadjuvant chemotherapy and/or radiotherapy. Similarly, our study showed that patients with metastatic ESCC and pretreatment lymphopenia had a shorter survival time than those without lymphopenia (8.2 months vs. 12.7 months; *P *=* *0.020).

We also analyzed the relationship between lymphopenia and tumor response to first‐line chemotherapy. The result showed that lymphopenia was significantly associated with lower ORR to chemotherapy (22.2% vs. 38.8%; *P *=* *0.045). The same result was found in several other tumors. Lissoni et al. 18 conducted a study that included 183 patients (lung cancer: 89 cases; colorectal cancer: 63 cases; breast cancer: 31 cases) and found that the ORR in lymphocytopenic patients was significantly lower than in patients with normal pretreatment lymphocyte counts (10% vs. 43%, *P *<* *0.001). Ceze et al. 10 also found that the objective response rate was significantly lower in lymphopenic patients than in other colorectal cancer patients receiving chemotherapy (12.5% vs. 40.2%; *P *=* *0.004).

There are several distinct mechanisms that chemotherapeutic agents can modify the interactions between tumor cells and host immune system 25. Chemotherapy succeeds in triggering tumor cell death, which restore or enhance the expression of tumor antigens and increase their susceptibility to immune attack 25, 26. In some cases, the immune system may contribute to make chemotherapy optimally efficient. Total lymphocyte counts play a key role in immune response. Lymphopenia might reflect a state of immune depression, which decreased the effect of immune attack causing the lower effect of therapy 18. However, the underlying mechanism between lymphopenia and decreased tumor response in ESCC patients is complicated and not fully revealed. Future studies need to be done to clarified the mechanisms in patients with metastatic ESCC.

Chemotherapy‐related toxicity was impacted by many factors, such as KPS, nutritional status, genetic polymorphism, and drug administration schedule. Our study showed that pretreatment lymphopenia was also related to increased risk of grade 3–4 hematological toxicity in ESCC patients. Several studies had demonstrated that in many tumors lymphopenia was associated with febrile neutropenia 17, platelet transfusion 27, and severe anemia 28. It was also found that early lymphopenia (day 5 after chemotherapy) was also a risk factor for chemotherapy‐induced febrile neutropenia 29. Furthermore, the subset of pretreatment lymphocytes, such as CD4+T cells, was considered to be an independent risk factor for febrile neutropenia 30.

Because pretreatment lymphocytes play a role in treatment‐related toxicity, as well as ORR and OS after first‐line chemotherapy, it was necessary to consider the influence of lymphocytes when selecting a treatment plan. For the patients with pretreatment lymphopenia, which indicated a low ORR and a high toxicity for chemotherapy, it was necessary to determine whether we could decrease the ration of chemotherapy and increase the ration of immunotherapy or targeted therapy.

The cause of pretreatment lymphopenia has not been fully clarified, and the explanations are likely to be multifactorial. Some studies demonstrated that tumors could directly induce T lymphocyte apoptosis using the Fas/FasL pathway 31. Overexpressed tumor‐derived antigens could cause the persistent polyclonal activation of lymphocytes, leading to their apoptosis 32. In addition to tumor‐related lymphopenia, host‐related factors could also lead to the decrease in lymphocytes. Altered lymphocyte homeostasis 33, such as a progressive decline of IL‐2 34 in the blood concentrations, appears to be associated with lymphopenia. Cachexia syndrome could exhibit lymphopenia, which might be due to the effect of cytokines, such as TNF‐α 35.

Previous studies 36, 37, 38 have demonstrated that surgery could induce immune suppression, by causing changes of lymphocyte numbers and subtypes. It was found that surgery and the associated neuroendocrine and paracrine responses could increase the secretion of immune suppressing hormones, which decreased numbers and activity of natural killer cells, Th1 and cytotoxic T lymphocyte cells and increased regulatory T cells, so as to reduce interleukin‐12 and interferon‐*γ* expression 37. However, those studies only focus on the immune response a short period of time after the surgery, the long‐term effects of surgery on host immune response in cancer patients are still uncertain. In our study, people who underwent primary tumor surgery had a higher proportion of lymphopenia than patients who did not undergo surgery (31.5% vs. 12.2%, *P* = 0.001). Due to the small numbers of patients with primary tumor surgery history in this retrospective study, we could not draw any definite conclusion; however, it is not possible to exclude that surgery can induce long‐term immune suppression and lymphopenia in some patients. Further prospective studies in metastatic ESCC patients are required to verify the results. Our results also showed that live metastasis, bone metastasis, and inflammatory factors (white blood cells, neutrophils, NLR, and PLR) were likely to be contributing factors because they were correlated with lymphopenia. These findings also indicated that lymphopenia was related to both tumor factors and host factors.

Based on these theories, some studies put forward the ideas of reversing lymphopenia or increasing the lymphocyte counts, expecting to improve survival and tumor response. Interventions in cancer patients, such as using hematopoietic stimulating factors, Toll‐like receptor (TLR) agonists, nutritional support, have showed promising results 39, 40, 41.

Our study also has several limitations. The retrospective approach may lead to inherent biases. Only those patients with collected pretreatment lymphocyte counts receiving first‐line chemotherapy at our institution were eligible for inclusion, possibly causing a selection bias. In the future, we will observe both the total and subset of peripheral blood lymphocyte counts, and the TILs can be evaluated in future studies.

In conclusion, pretreatment blood lymphocyte count was an easily detectable predictive factor for stage IV ESCC patients receiving first‐line chemotherapy. Pretreatment lymphopenia indicated short‐term survival, as well as a low tumor response and high treatment‐induced toxicity. Our results, combined with future analyses of subtype and the dynamic change in lymphocyte counts, may lead to individualized treatment and provide evidence for future immunotherapy of ESCC.

## Conflict of Interest

None declared.
